# A Review of RRx-001: A Late-Stage Multi-Indication Inhibitor of NLRP3 Activation and Chronic Inflammation

**DOI:** 10.1007/s40265-023-01838-z

**Published:** 2023-03-15

**Authors:** Nanthini Jayabalan, Bryan Oronsky, Pedro Cabrales, Tony Reid, Scott Caroen, Aishwarya M. Johnson, Natalia A. Birch, John D. O’Sullivan, Richard Gordon

**Affiliations:** 1 Faculty of Medicine, UQ Centre for Clinical Research (UQCCR), The University of Queensland, Brisbane, 4029 Australia; 2 EpicentRx, Torrey Pines, CA 92307 USA; 3 UCSD Department of Bioengineering, La Jolla, CA 92093 USA; 4 Centre for Microbiome Research, School of Biomedical Science, Translational Research Institute, Queensland University of Technology, Brisbane, 4102 Australia; 5 Department of Veterinary Medicine College of Food and Agriculture, Emirates University, Al Ain, UAE

## Abstract

Chronic unresolving inflammation is emerging as a key underlying pathological feature of many if not most diseases ranging from autoimmune conditions to cardiometabolic and neurological disorders. Dysregulated immune and inflammasome activation is thought to be the central driver of unresolving inflammation, which in some ways provides a unified theory of disease pathology and progression. Inflammasomes are a group of large cytosolic protein complexes that, in response to infection- or stress-associated stimuli, oligomerize and assemble to generate a platform for driving inflammation. This occurs through proteolytic activation of caspase-1-mediated inflammatory responses, including cleavage and secretion of the proinflammatory cytokines interleukin (IL)-1β and IL-18, and initiation of pyroptosis, an inflammatory form of cell death. Several inflammasomes have been characterized. The most well-studied is the nucleotide-binding domain (NOD)-like receptor protein 3 (NLRP3) inflammasome, so named because the NLRP3 protein in the complex, which is primarily present in immune and inflammatory cells following activation by inflammatory stimuli, belongs to the family of nucleotide-binding and oligomerization domain (Nod) receptor proteins. Several NLRP3 inflammasome inhibitors are in development, all with multi-indication activity. This review discusses the current status, known mechanisms of action, and disease-modifying therapeutic potential of RRx-001, a direct NLRP3 inflammasome inhibitor under investigation in several late-stage anticancer clinical trials, including a phase 3 trial for the treatment of third-line and beyond small cell lung cancer (SCLC), an indication with no treatment, in which RRx-001 is combined with reintroduced chemotherapy from the first line, carboplatin/cisplatin and etoposide (ClinicalTrials.gov Identifier: NCT03699956). Studies from multiple independent groups have now confirmed that RRx-001 is safe and well tolerated in humans. Additionally, emerging evidence in preclinical animal models suggests that RRx-001 could be effective in a wide range of diseases where immune and inflammasome activation drives disease pathology.

## Key Points

**Table:** 

The nucleotide-binding domain (NOD)-like receptor protein 3 (NLRP3) inflammasome is a large protein complex that kickstarts the inflammatory response.
Persistent activation of NLRP3 leads to chronic inflammation, a common denominator of several diseases and conditions.
This review focuses on RRx-001, a well-tolerated NLRP3 inhibitor that targets chronic inflammation and that is currently in a phase 3 clinical trial for the treatment of late-stage cancer.

## Introduction

**Fig. 1:**
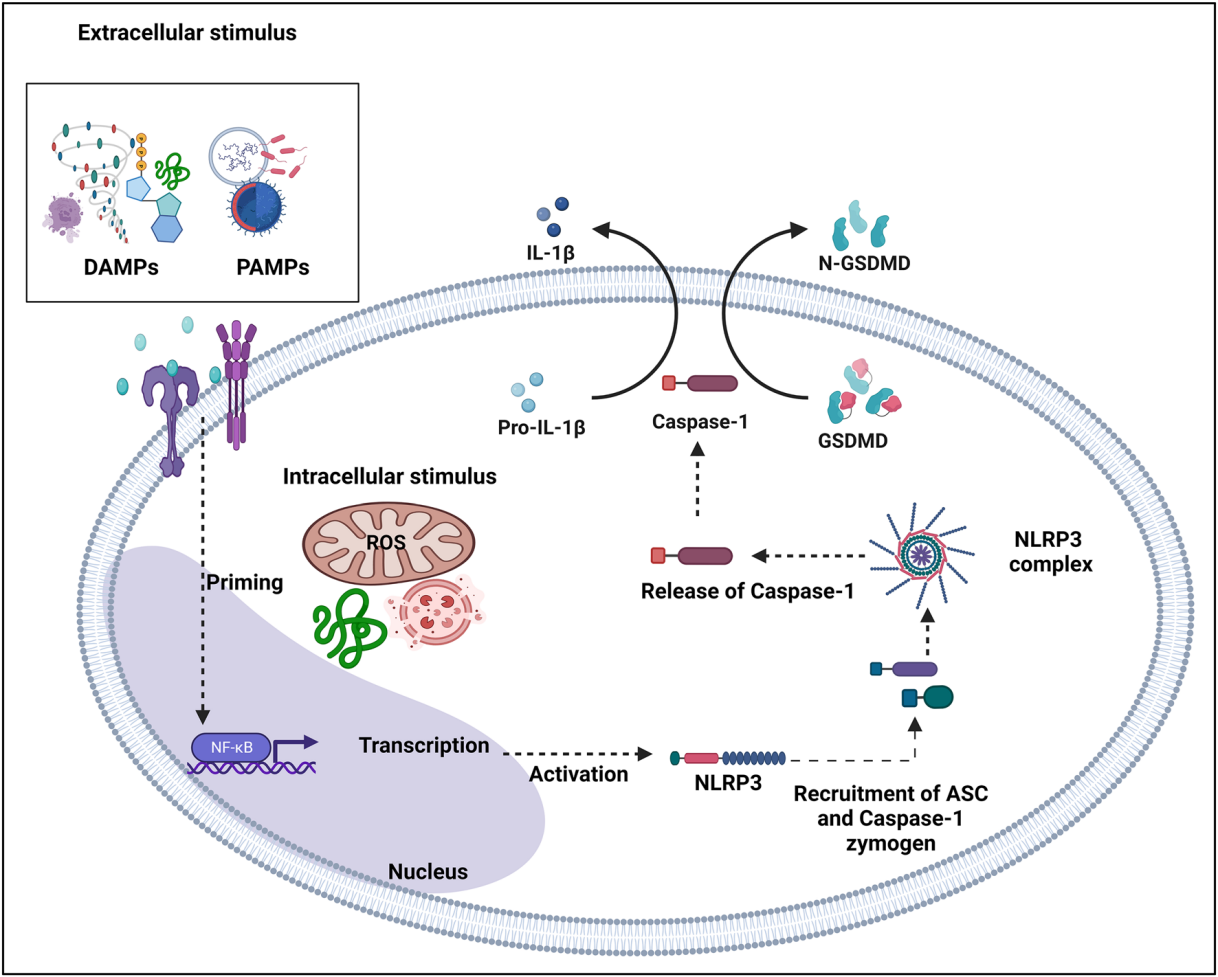
Schematic of inflammasome activation in immune cells. A range of PAMPs and DAMPs, including bacterial and viral proteins, elevated intracellular ROS levels, ATP, nucleic acid ligands, uric acid crystals, cholesterol crystals, and silica activate inflammasomes. Activated inflammasomes cleave caspase-1 according to the NF-κB-dependent (NF-κB not shown) canonical pathway or caspase-4/5 according to the NF-κB-dependent (NF-κB not shown) non-canonical pathway. These activated caspases, in turn, cleave GSDMD to form the N-fragment of GSDMD and cell membrane pores, initiating pyroptosis. Activated caspase-1 also cleaves pro-IL-1β and pro-IL-18 into IL-1β and IL-18, which are released through the gasdermin N pores, promoting inflammation [4]. *ASC* apoptosis-associated speck-like protein, *ATP* adenosine triphosphate, *DAMPs* danger-associated molecular patterns, *GSDMD* gasdermin D, *IL* interleukin, *LRR* leucine-rich repeats, *NF-κB* nuclear factor-κB, *NLRP3* NOD-, LRR-, and pyrin domain-containing 3, *NOD* nucleotide-binding domain, *PAMPs* pathogen-associated molecular patterns, *ROS* reactive oxygen species

At the heart of physics is a paradox between what is directly observable and what is not, between general relativity and quantum mechanics, which Einstein, for all his considerable genius, could never reconcile, despite decades of his life spent in pursuit of a unified theory [1]. Perhaps the most promising candidate to bridge the gap between the relativistic and the quantum realms is so-called string theory, which, however, is not universally accepted as a cosmological model [2]. A similar theory of everything (TOE) is provided in medicine by chronic inflammation, which underlies, initiates, or contributes to many, if not all, diseases. Central to the inflammatory process is priming and activation of inflammasomes, which are large cytosolic protein complexes found in macrophages, dendritic cells, and other immune and non-immune cells. In response to infection- or stress-associated stimuli, inflammasomes oligomerize and self-assemble to induce proteolytic cleavage and secretion of the proinflammatory cytokines interleukin 1-beta (IL-1β) and interleukin-18 (IL-18), and initiate pyroptosis or caspase-1-dependent lytic cell death [3], as outlined in Fig. 1.

Accordingly, the purpose of a class of medications known as inflammasome inhibitors is to “extinguish” or suppress inflammasome activation, which drives persistent low-grade inflammation, and which is now considered to be a pathological hallmark of most chronic diseases. Like several steroidal and non-steroidal anti-inflammatory agents that have been successfully developed to resolve the five cardinal hallmarks of inflammation [5], *tumor* (swelling), *dolor*, (pain), *rubor*, (redness), *calor* (heat), and *functio laesa* (loss of function), inflammasome inhibitors are emerging as promising therapeutic agents to target and resolve the vicious cycle of chronic immune activation and disease progression.

Amongst the multiple inflammasomes that have been characterized to date, including those from the nucleotide-binding and oligomerization domain (Nod) and leucine-rich repeat-containing (NLR) family, by far the most well-studied is the nucleotide-binding domain (NOD)-like receptor protein 3 (NLRP3) inflammasome, which activates in response to a wide range of danger- and pathogen-associated molecular patterns (DAMPs and PAMPs, respectively) [6]. The NLRP3 inflammasome is a three-component platform with a sensor, NLRP3, an adaptor, apoptosis-associated speck-like protein (ASC), and a pro-caspase-1 zymogen effector that drives pyroptosis, a rapid, lytic form of cell death, and proteolytic maturation of the proinflammatory cytokines IL-18 and IL-1β, as illustrated in Fig. 1 [7]. This pathway is classified as nuclear factor-κB (NF-κB)-dependent “canonical NLRP3 inflammasome activation”. In addition, an NF-κB-dependent “non-canonical NLRP3 inflammasome activation”, which involves caspase-11 in mice and caspase-4/5 in humans, has been described [8]. The NLRP3 protein contains three domains: NACHT, a central nucleotide domain with ATPase activity, C- terminal leucine-rich repeat (LRR), which recognizes DAMPs and PAMPs, and an N-terminal pyrin domain (PYD) that binds to ASC [9].

The activation mechanism of NLRP3 involves a priming step and an activation step [10]. The priming step involves the triggering of Toll-like receptors (TLRs) by their ligands such as PAMPs, causing the translocation of NF-κB to the nucleus, which in turn enhances the transcription and increases protein levels of NLRP3 and pro-IL-1β [11]. Once primed, the activation of NLRP3 inflammasome is initiated by an array of stressors such as adenosine triphosphate (ATP), misfolded proteins, PAMPs, and DAMPs, which facilitate the oligomerization of inactive NLRP3 and binding to the adaptor protein ASC through a PYD–PYD interaction [12]. This recruits procaspase-1, which gets cleaved to active caspase-1, the effector protein. Active caspase-1 cleaves pro-IL-1β and pro-IL-18 into their mature and biologically active form as well as gasdermin D (GSDMD) [13, 14]. The newly formed N-terminal fragment of GSDMD inserts into the plasma membrane and forms pores, leading to leakage of cellular contents and, ultimately, pyroptosis [15].

A Pandora’s Box of chronic diseases is associated with chronic and unresolving NLRP3 inflammasome activation, including multiple sclerosis, Alzheimer’s disease, Parkinson’s disease, stroke, cancer, atherosclerosis, type 2 diabetes, gout, inflammatory bowel disease (IBD), coronavirus disease 2019 (COVID-19), pulmonary hypertension, heart disease, non-alcoholic steatohepatitis (NASH), etc. This potentially renders inflammasome inhibitors a disease-modifying treatment for a wide range of conditions or diagnoses, which is not so far-fetched or unrealistic, considering that pro-inflammatory checkpoint inhibitors (immune checkpoint inhibitors [ICIs]), essentially the mirror image of the inflammasome inhibitors, have also been touted as “magic bullets” and “cure-alls,” theoretically applicable to all cancers (which, by consensus, are not one disease but many separate diseases) [16], especially in combination with other immunotherapies [17, 18]. The recent clinical ubiquity of ICIs attests to the central importance of inflammation in multifactorial conditions such as cancer, sepsis [19], and viral infections [20]. For this reason, inflammation has been referred to as a “necessary evil” [21, 22]—necessary because acute, self-limited inflammatory responses are protective against infection, irritation, and injury, whereas unresolving chronic inflammation is harmful and exacerbates ongoing pathology. Accordingly, the defining characteristic of many different diseases, such as arthritis, connective tissue disorders, cancer, chronic obstructive pulmonary disease (COPD), diabetes, and heart failure, is chronic, persistent inflammation [23].

Extending a popular analogy from “Goldilocks and the Three Bears” applied to the optimal distance of a planet from its sun to be able to sustain life, termed the Goldilocks zone, the emerging evidence indicates that an optimal range, timing, and duration exists for inflammatory responses to maintain health and longevity. The harmful effects of an excessive short-term inflammatory “*storm*” has been starkly demonstrated in COVID-19-related mortality during the ongoing pandemic [24], whereas the paradigm of chronic unresolving inflammation, which contributes to and exacerbates a range of inflammatory and autoimmune diseases, is now well-established [25]. Exactly what constitutes too much or too little inflammation and the degree of harm that results (or that may result) from either are largely context dependent and vary according to factors such as age, tissue-specific stimuli, genetic traits, the type of environmental stress, and chronicity [26].

## RRx-001, a Clinical Stage Direct Covalent NLRP3 Inhibitor Targeting Multiple Immune and Inflammatory Pathways Involved in Chronic Inflammation

As expertly discussed in several major reviews on the current status of inflammasome-targeted therapeutics [27, 28], multiple “disease agnostic” NLRP3 inflammasome inhibitors are in development for systemic and central nervous system (CNS) diseases [29–32]. The most clinically advanced of these direct NLRP3 inhibitors include a sulfonylurea class of drugs such as MCC950 and its derivatives [33], developed by Inflazome and acquired by Roche, dapansutrile/OLT1177 from Olatec Therapeutics [34], and RRx-001 from EpicentRx. The primary focus of this review is RRx-001 (generic name bromonitrozidine) and its therapeutic potential for NLRP3-driven inflammatory diseases. The other advanced NLRP3 inhibitors such as MCC950, OLT177, and others have been comprehensively discussed elsewhere in recent reviews [35, 36].

RRx-001 is a potent and patent-protected NLRP3 inflammasome inhibitor that was derived from trinitroazetidine (TNAZ), having been co-discovered with a leading United States aerospace manufacturer; it was rendered less impact sensitive and more reactive with cysteines by the replacement of a nitro group from TNAZ with an acyl bromide moiety [37]. This modified and safer version, RRx-001, has been extensively studied in vitro and in animal models of several inflammatory and protective indications including Alzheimer’s disease, stroke, multiple sclerosis, oral mucositis, NASH, IBD, and chemo- and radioprotection. However, as cancer represented the initial primary development path for RRx-001, the anti-inflammatory and normal tissue protective properties have not been comprehensively discussed in the literature. This review summarizes the mechanism of action and multi-model therapeutic potential of RRx-001 as an inhibitor of the NLRP3 inflammasome pathway at both the key priming and activation stages.

RRx-001 is the most clinically advanced of the current NLRP3 inflammasome inhibitors in terms of human testing studies and safety, having been safely evaluated in over 300 comorbid, treatment-resistant cancer patients in 12 clinical trials, including in an ongoing phase 3 trial for the treatment of small cell lung cancer (SCLC) (REPLATINUM; NCT03699956) [38]. SCLC was chosen as a phase 3 indication because it is a recalcitrant tumor type where resistance is the rule and not the exception and in phase 2 RRx-001 demonstrated that it not only resensitized resistant SCLC to platinum-based chemotherapy but also reduced platinum-related myelotoxicity [38, 39]. In addition, a late-stage trial is planned with RRx-001 in head and neck cancer as a treatment for chemotherapy- and radiation-induced severe oral mucositis (SOM). The main, and arguably the only, toxicity of RRx-001 is a sterile painful infusion phlebitis. This toxicity is largely mitigated when RRx-001 is co-administered with a 10-cc aliquot of autologous blood [40]. Compared with placebo, no increase in infections has ever been observed with RRx-001 both when it is administered alone or in combination with chemotherapy. Despite binding to red blood cells, RRx-001 is not associated with anemia. Besides NLRP3 inhibition, activity of RRx-001 in cancer depends on the convergence of multiple mechanisms, including macrophage polarization [41], CD47 antagonism [42], vascular normalization [43], and epigenetic modification [44]. RRx-001 is also on the critical path for approval from the Food and Drug Administration’s (FDA’s) Animal Rule as a radiation medical countermeasure in case of accidental radiological exposure from a nuclear attack or nuclear reactor meltdown. The Animal Rule substitutes well-controlled animal studies for human studies in cases where human studies are infeasible and/or unethical, as in this case, when exposure to high-dose ionizing radiation is required [45].

## Inflammasome and Anti-inflammatory Mechanisms of Action

As an electrophile, RRx-001 selectively and covalently targets deprotonated (−S^−^), nucleophilic thiolates with a low acid dissociation constant (p*K*a) in specific proteins [46], including cysteine 409 of the NLRP3 inflammasome and the beta cysteine 93 residue on hemoglobin [47]. The lower the p*K*a value, which favors the formation of the thiolate anion, the more reactive the cysteine with RRx-001. The presence of basic, readily protonated amino acids in proximity to a cysteine, such as cysteine 409 of the NLRP3 inflammasome and beta cysteine 93 on hemoglobin, decreases the p*K*a and thus increases its reactivity with RRx-001.

**Fig. 2:**
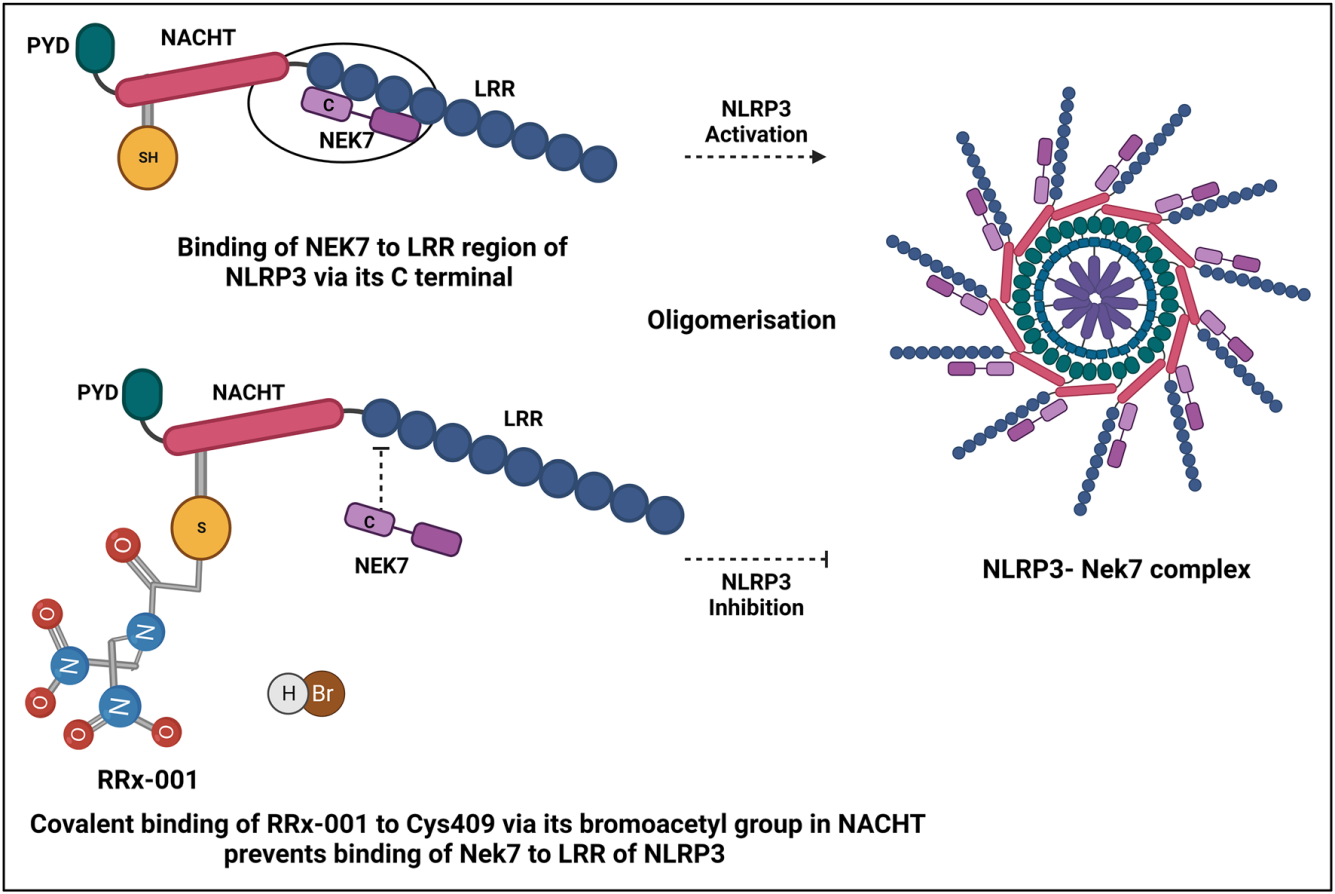
Inhibition of NLRP3 inflammasome activation by RRx-001 in immune cells. Binding and interaction of NEK7 with the C-terminal LRR domain of NLRP3 is required for oligomerization and assembly of the inflammasome complex to proceed. Covalent binding of RRx-001 to Cys409 in the adjacent NLRP3 NACHT domain via its bromoacetyl group prevents the interaction and binding NEK7 in the C-terminal LRR domain of NLRP3, thus inhibiting inflammasome complex assembly and activation in immune cells. *NLRP3* NOD-, LRR-, and pyrin domain-containing 3, *NEK7* NIMA-related kinase 7, *LRR* leucine-rich repeats, *Cys* cysteine, *NACHT* domain conserved in *NAIP* Neuronal apoptosis inhibitory protein, *CIITA* MHC class II transcription activator, *HET-E* Incompatibility locus protein from Podospora anserina and *TP1* Telomerase-associated protein

As outlined in Fig. 2, the covalent interaction between NLRP3 cysteine 409 located in its NACHT domain and RRx-001 via its bromoacetyl group prevents the critical interaction between NLRP3 and NIMA-related kinase 7 (NEK7), which is required for oligomerization and assembly of the NLRP3 inflammasome complex. This critical step is essential for downstream recruitment of caspase-1 and autoproteolytic cleavage into its active heterodimeric complex of p20/p10 or p33/p10, which initiates processing and release of active IL-1β, IL-18, and ASC followed by pyroptotic cell death. This capase-1-dependent process is known as “canonical” inflammasome activation.

A non-canonical inflammasome activation pathway involves the oligomerization of caspase-11 in mice or caspase-4/5 in humans, which binds to cytosolic lipopolysaccharide (cLPS), where the activation of caspases induces inflammatory responses [48]. The non-canonical inflammasome pathway activates caspase-1 via the NLRP3 inflammasome pathway [49]. RRx-001 was shown to be effective in blocking NLRP3 activation in response to diverse upstream triggers including ATP, uric acid, and nigericin, with nanomolar potency in these studies (IC_50_ =116.9 nM)[50]. RRx-001 specifically inhibits canonical and non-canonical NLRP3 inflammasome activation in both mouse and human macrophages in vitro, but not the NLR family, pyrin domain-containing 1 (NLRP1), or Nod-like receptor family card containing 4 (NLRC4) and in melanoma 2 (AIM2) inflammasomes. The half-life was dependent on the inflammasome turnover rate. This indicates that RRx-001 is a direct NLRP3 inflammasome inhibitor. In addition, other independent studies have indicated that RRx-001 significantly inhibits lipopolysaccharide (LPS)-induced activation of (TAK1) Transforming growth factor-β-activated kinase 1, an upstream regulator of the NF-κB and mitogen-activated protein kinase (MAPK) pathways, which are involved in priming of the NLRP3 inflammasome, which is required in some cell types [51].It is possible that RRx-001 binds to components of NLRP3 such as the adaptor or effector protein even before the formation of the NLRP3 inflammasome components as a manner to inhibit the NLRP3 inflammasome activation. However, this warrants future research.

**Fig. 3:**
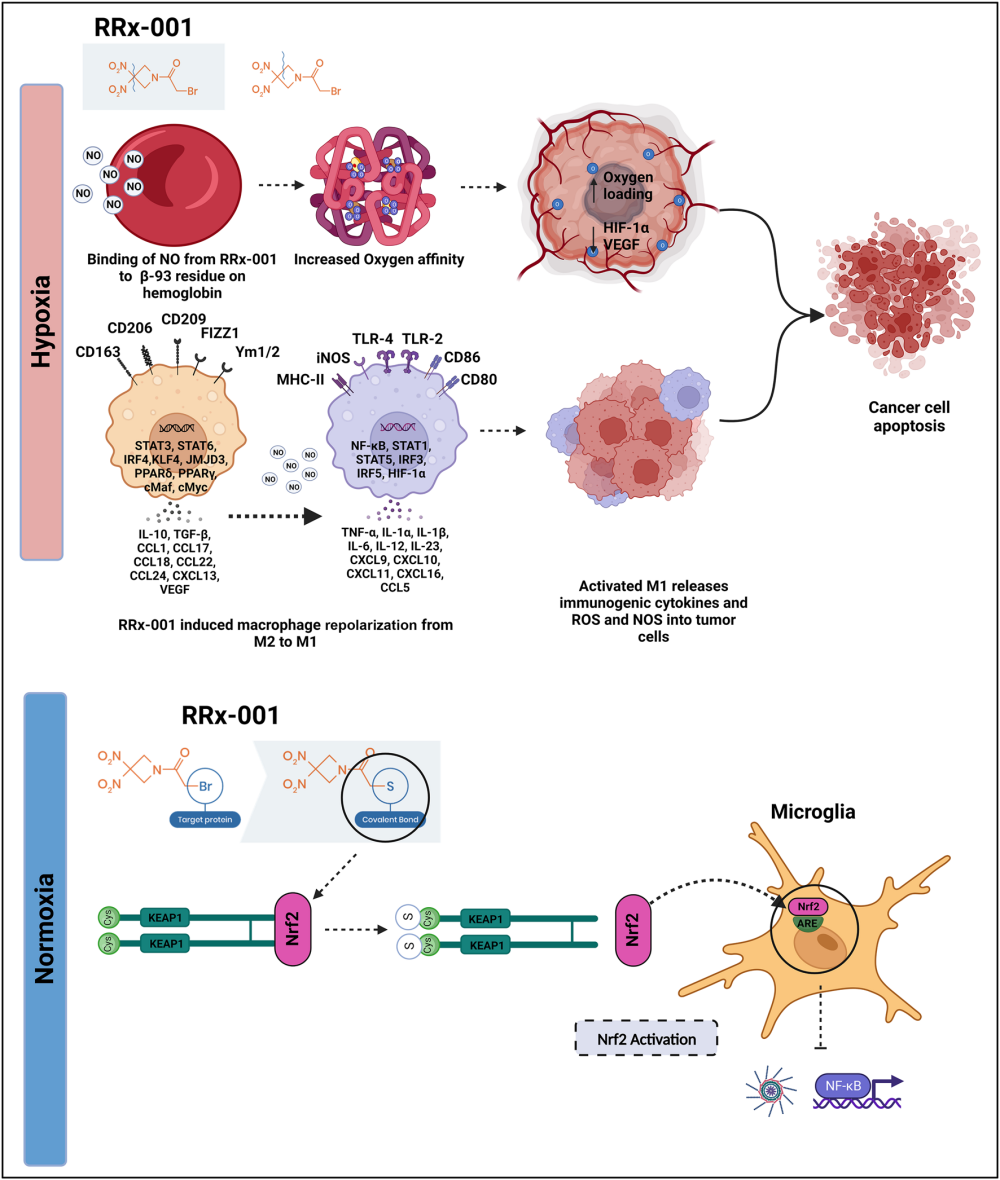
Therapeutic activity of RRx-001 during hypoxic and normoxic conditions. Under hypoxic conditions found in tumors, RRx-001 fragments function as an NO donor in red blood cells, increasing oxygen loading and increasing cancer cell apoptosis. RRx-001 also triggers macrophage repolarization from the M2 to M1 phenotype, which generates immunogenic cytokines, ROS, and NOS in hypoxic tumors. Under normoxic conditions found in most inflammatory diseases, RRx-001 functions as an inhibitor of the NLRP3 and NF-κB pathways and an activator of the Nrf2 antioxidant pathway. These work in concert to block priming and activation of the NLRP3 inflammasome. *ARE* antioxidant responsive element, *CCL* chemokine (C-C motif) ligand, *cMaf* cellular musculoaponeurotic fibrosarcoma oncogene homolog, *cMyc* cellular myelocytomatosis  *Cys* cysteine, *CXCL* chemokine (C-X-C motif) ligand, *FIZZ* found in inflammatory zone, *HIF* hypoxia inducible factor, *IL* interleukin, *iNOS* inducible nitric oxide synthase, *IRF* interferon-regulatory factor, *JMJD* Jumonji C domain-containing protein, *KEAP* Kelch-like ECH-associated protein, *KLF* Krüppel-like factor, *LRR* leucine-rich repeats, *MHC* major histocompatibility complex, *NF-κB* nuclear factor-κB, *NLRP3* NOD-, LRR-, and pyrin domain-containing 3, *NO* nitric oxide, *NOD* nucleotide-binding domain, *NOS* nitrogen oxide species, *Nrf2* nuclear factor erythroid 2-related factor 2, *PPAR* peroxisome proliferator-activated receptors, *ROS* reactive oxygen species, *STAT* signal transducer and activator of transcription, *TGF* transforming growth factor, *TLR* Toll-like receptor, *TNF* tumor necrosis factor, *VEGF* vascular endothelial growth factor

Also relevant to the anti-inflammatory effects of RRx-001, besides NLRP3 and NF-κB pathway inhibition, is its activation of nuclear factor erythroid 2-related factor 2 (Nrf2). This transcription factor increases the expression of several antioxidant enzymes such as superoxide dismutase (SOD), catalases (CATs), thioredoxins (Trxs), peroxiredoxins (Prxs), reductases, and peroxidases to protect against damage from oxidative stress, which regulates and sustains multiple inflammatory pathways including NLRP3 [52]. The transcriptional activity of Nrf2 is mainly held in check by a redox-sensitive repressor called Keap1 (Kelch-like ECH-associated protein) [53]. RRx-001-mediated Nrf2 activation is likely related to modification of a key Keap1 cysteine residue, cysteine 151, which disrupts the Keap1–Nrf2 complex and results in constitutive activity of Nrf2. In addition, under hypoxic conditions, RRx-001 fragments lead to the release of nitric oxide, as illustrated in Fig. 3.

### Mechanisms of NLRP3 Inhibition by RRx-001 in Cancer

RRx-001 interacts and inhibits multiple therapeutic targets such as Myc [54], Keap1 [55], and NLRP3 [50] via its interaction with cysteine residues. However, RRx-001 has a very high potency for NLRP3. This was demonstrated by Chen et al. [50], where in a normoxic environment, the non-fragmented RRx-001 binds covalently to cysteine 409 of NLRP3 via its bromoacetyl group and therefore blocks the NLRP3–NEK7 interaction, and in a hypoxic malignancy environment, RRx-001 fragments [56]. The resulting moieties have a plethora of anticancer effects that are only induced within the cancer environment. However, the mechanistic action of RRx-001 in inhibiting the NLRP3 inflammasome in cancer is unclear. Here, we have described the possible mechanistic action of RRx-001 in inhibiting NLRP3 in cancer models. The heightened levels of IL-1β are associated with cancer progression. However, activation of the NLRP3 inflammasome promotes the release of IL-1β, which not only creates a pro-inflammatory environment but also has profound effects on cancer cell proliferation, migration, and metastasis [57]. Additionally, the activation of macrophages plays a crucial role for the secretion of IL-1β. Thus, it is possible, in a cascade manner, the binding of RRx-001 to NLRP3 inhibits the polarization of macrophage and thus reduces the release of IL-1β, where programmed death-ligand 1 (PD-L1) acts as an ICI that regulates the immune cells, preventing heightened immune responses and autoimmunity. It is proven that cancerous cells exploit this characteristic mechanism by overexpressing PD-L1 on the cell surface, and several studies are targeting PD-1/PD-L1 for cancer therapy [58]. Interestingly, Theivanthiran et al. points towards a mechanistic link between PD-L1 and NLRP3 activation as a possible therapeutic intervention in immunotherapy [59]. Hence, further studies with NLRP3 inhibitors such as RRx-001 will help elucidate the PD-L1/NLRP3 signaling axis in immune-related adverse effects. Overall, further study is needed to elucidate the exact mechanisms associated with NLRP3 inhibition by RRx-001 in malignancies.

## RRx-001 Activity in Chemo and Radioprotective Indications

Common to chemotherapy and radiation is the induction of damage to both normal tissues and tumors through mechanisms that involve ROS generation, and the NLRP3 inflammasome. Several radio- and/or chemoprotectors have been investigated in experimental studies to improve the therapeutic ratio of these treatments, but to date, only one, amifostine, has been approved. However, due to severe adverse events, including nausea, emesis, hypotension, and hypokalemia, as well as potential evidence that it inappropriately protects tumors, amifostine is not used in the clinic [60]. In preclinical and clinical studies, presented below, RRx-001 has demonstrated evidence of chemoradioprotection. See Tables 1 and 2 for a summary.

### Mucositis

SOM is an inflammatory mucosal lesion, which manifests during treatment with radiotherapy and cisplatin for head and neck cancers [61]. In a randomized, 53-patient, clinical trial called PREVLAR (ClinicalTrials.gov Identifier: NCT03515538), which compared RRx-001 + standard-of-care (SOC) cisplatin and intensity-modulated radiation therapy (IMRT) to SOC cisplatin and IMRT alone, RRx-001-treated patients experienced SOM to a degree that was less severe and of shorter duration and demonstrated delayed time to onset and resolved earlier in comparison to SOC alone [56].

### In Vivo Chemoprotection of the Kidneys and Bone Marrow

To determine the effect of RRx-001 pretreatment on cisplatin-induced bone marrow suppression and renal toxicity, the following experiment was conducted: BALB/c mice were divided into three groups: (1) no treatment, (2) vehicle and cisplatin only, and (3) RRx-001 and cisplatin. RRx-001 treatment (5 mg/kg every other day for 3 days) was initiated 3 days prior to cisplatin administration. The results demonstrated that RRx-001 pretreatment significantly (*p* < 0.05) decreased the blood urea nitrogen and creatinine levels. A statistically significant (*p* < 0.05) reduction in the mean total chromosome aberration frequency per metaphase in the RRx-001 and cisplatin group compared to the cisplatin-only group was also observed [62].

### Gastrointestinal (GI) Radioprotection

Normal C3H mice intraperitoneally treated with 10 mg/kg RRx-001 or with vehicle were irradiated with total body irradiation (TBI) at a single dose of 10–15 Gy at a dose rate of 60 cGy/min. Three days following TBI, mice were euthanized, and samples of the duodenum, jejunum, and ileum were immediately removed, fixed, and stained with hematoxylin and eosin (H&E). Surviving gastrointestinal crypts or stem cells were counted by light microscopy. At 10 mg/kg, RRx-001 dosed 30 min before TBI did not sensitize crypt cells to radiation and significantly increased the crypt survival compared to TBI alone [63, 64].

### Survival After Lethal Irradiation

To determine the survival efficacy of RRx-001 treatment against radiation doses that cause the hematopoietic subsyndrome of acute radiation syndrome (ARS), CD2F1 mice were treated with a lethal 70/30 whole body irradiation dose of 9.35 Gy at 0.6 Gy/min using a ^60^Co source. Twenty-four hours prior to irradiation, all mice were intraperitoneally injected with either 10 mg/kg RRx-001 or the vehicle control (5% dimethyl sulfoxide in sterile water). Survival improvement in favor of pretreatment with one dose of 10 mg/kg RRx-001 over vehicle control in irradiated mice was highly significant (*p* < 0.0005), with an approximate 33.4% reduction in the 30-day death risk [65].

### GI Chemoprotection

In a phase 1 trial named PAYLOAD (NCT02801097), RRx-001 was co-administered with irinotecan in 12 treatment-resistant cancer patients that had previously received irinotecan, a chemotherapy agent, which is associated with a 20–35% rate of severe diarrhea (≥ grade 3). In PAYLOAD, no grade 2 or above diarrhea was observed with irinotecan treatment, which accords with clinical experience in another trial, ROCKET (NCT02096354), where 34 patients with third/fourth-line colorectal cancer were randomized 2:1 to receive RRx-001 + irinotecan or the current SOC, regorafenib. None of the RRx-001 randomized patients in ROCKET experienced > grade 1 diarrhea [66].

## RRx-001 in Cardiovascular, Cardiopulmonary, and Metabolic Diseases

### Cardioprotection

A total of 24 BALB/c mice were randomized to prophylactic treatment with vehicle, RRx-001, or no-intervention control. Within each of the three intervention arms, mice received treatment with doxorubicin, the main adverse event of which is cardiotoxicity. Murine pressure-volume (PV) analysis was performed with microconductance catheters to characterize the degree of cardiovascular dysfunction within each group. The hemodynamic parameters left ventricular systolic pressure (LVSP), heart rate, and maximal rate of increase of left ventricular pressure (± dP/dt max), all significantly improved (*p* < 0.05) in RRx-001-treated mice compared with untreated doxorubicin mice [67].

### Enhancement of Exercise Performance

Mice subjected to forced running after three once weekly intravenous doses of 2 and 5 mg/kg RRx-001 or vehicle demonstrated significantly increased exhaustive exercise times in the RRx-001 groups compared to control. In addition, serum IL-1β was significantly lower in the RRx-001 groups than in the vehicle group (*p* < 0.05), as were serum creatinine kinase levels (*p* < 0.05) [68].

### Ischemia Reperfusion Injury

Reperfusion after ischemia generates oxidative stress and inflammation. Ischemia reperfusion (I/R) injury was studied in a hamster chamber window, with compression of the periphery of the window for 1 h to induce ischemia. Animals received intravenous RRx-001 (5 mg/kg) 24 h before ischemia, and sodium nitrite (10 nmols/kg) was supplemented 10 min after the start of reperfusion. Vessel diameter, blood flow, adherent leukocytes, and functional capillary density were assessed by intravital microscopy at 0.5, 2, and 24 h following the release of the ischemia. The results demonstrated that, compared to control, RRx-001 preconditioning increased blood flow and functional capillary density, and preserved tissue viability in the absence of side effects over a sustained time [69].

### Hemorrhagic Shock

The gold standard for resuscitation from hemorrhagic shock is transfusion with blood, which is often in short supply. Hence, RRx-001 was tested as a replacement for blood transfusion. Hamsters that underwent severe hemorrhage and that remained in hypovolemia for 1 h received volume resuscitation by an infusion of 25% of fresh blood or fresh blood treated with RRx-001 (140 mg/kg). Compared to resuscitation with blood alone, animals treated with RRx-001 experienced decreased vascular resistance, increased blood flow, and functional capillary density immediately after resuscitation, with preserved tissue viability. Furthermore, in RRx-001-treated animals, mean arterial pressure (MAP) was maintained within normal levels after resuscitation (MAP > 90 mmHg) [70].

### Cerebral Malaria

In an experimental mouse model of cerebral malaria (ECM), 10 mg of RRx-001 given intravenously every other day for 3 days demonstrated anti-parasitic activity and significantly preserved cerebral perfusion and reduced the number of adhered and rolling leukocytes, alone or combined with the antimalarial agent artemether [71].

### Asthma

In an inhaled house dust mite (HDM) mouse model of asthma, where 10 μg HDM was intranasally instilled in C57BL/6J mice on days 0 and 7–11, intraperitoneal treatment with 10 mg/kg RRx-001 on days 7, 9, and 11 significantly reduced inflammatory cell infiltration and mucus secretion in the airway [72].

### Pulmonary Hypertension

The hallmark features of pulmonary artery hypertension (PAH) are vascular remodeling, elevated pulmonary blood pressure, oxidative stress, and right ventricular hypertrophy. In male Wistar rats (180–220 g), RRx-001 significantly attenuated hypoxia-induced increases in right ventricular systolic pressure (RVSP) and right ventricular hypertrophy (RVH) in PAH rats versus vehicle. RRx-001 also significantly decreased the thickening of pulmonary artery media and decreased the muscularization of pulmonary arterioles in PAH rats and the wall thickness/external diameter ratio of the pulmonary arteries versus vehicle [73].

## Efficacy of RRx-001 in Central Nervous System Diseases

RRx-001 has demonstrated activity in several neurodegenerative disorders, including multiple sclerosis and Alzheimer’s disease, in which an important contributor and common denominator is chronic neuroinflammation.

### Alzheimer’s Disease

The neuroprotective effects of 2 mg/kg RRx-001 administration to Alzheimer's disease (AD), which has been linked to aberrant NLRP3 activation, were evaluated via intraperitoneal injection once weekly for 3 months in aged (21- to 24-month-old) triple transgenic Alzheimer’s disease model (3×Tg-AD) mice and control non-transgenic (NonTg) mice. RRx-001-treated mice demonstrated improved performance on learning and spatial memory tasks and greater risk assessment behavior based on the elevated plus maze test. A significant increase in reduced glutathione and a decrease in lipid peroxidation and amyloid plaque density were also observed in the 3×Tg-AD mice, suggesting that RRx-001 reverses histological hallmarks of Alzheimer’s disease and protects cognitive and emotional function in aged 3×Tg-AD mice [74].

### Multiple Sclerosis

In an experimental autoimmune encephalomyelitis (EAE) model of multiple sclerosis, C57BL/6J mice intraperitoneally injected with RRx-001 demonstrated reduced inflammatory infiltration and demyelination in the spinal cord compared with mice in the vehicle control group. In addition, RRx-001 treatment was associated with decreased CNS expression levels of the proinflammatory cytokines IL-l8, IL- 6, and tumor necrosis factor (TNFα). In NLRP3 knockout mice, no effect was observed with RRx-001 on the clinical course of EAE or on the infiltration of immune cells in the CNS, which suggests that RRx-001 ameliorates EAE through NLRP3 inhibition.

## RRx-001 in Intestinal Inflammation and Inflammatory Bowel Disease

IBD, a rising global health problem, which encompasses Crohn’s disease and ulcerative colitis, is associated with uncontrolled inflammasome activation [75]. In dextran sulfate sodium (DSS)-induced colitis, a mouse model of human IBD, mice were treated with 3% DSS dissolved in the drinking water for 6 days to induce IBD. RRx-001 (5, 10 mg/kg) or vehicle was administered daily through the intraperitoneal route for 10 days. RRx-001 treatment markedly ameliorated weight loss, disease activity, and colon shortening, a macroscopic pathological feature of colitis. Histologically, RRx-001 treatment decreased the destruction of the intestinal epithelium and infiltration of immune cells. Also, the level of IL-1β in serum was decreased, suggesting an inhibitory effect on the NLRP3 inflammasome in vivo.

## Discussion and Conclusion

Inflammation, especially when it is chronic or persistent, has been implicated as a central underlying cause of many, if not most, diseases through the infliction of collateral damage on otherwise healthy cells in tissues. This raises the scenario of almost unrestricted use with NLRP3 inflammasome inhibitors, which smacks of implausibility, considering that the classic anti-inflammatory agents, glucocorticoids, colchicine, and cyclooxygenase (COX-1 and COX-2) inhibitors, are far from universally prescribed or universally effective, in part because of severe toxicities.

Certainly, RRx-001, despite a tolerable safety profile and what appears to be a favorable risk/benefit ratio, has been met with skepticism and resistance for not staying in its anticancer lane and for “trying to do too much.” This is the distinct dilemma with all NLRP3 inflammasome inhibitors: exactly which therapeutic indication to choose when so many are available to choose from, as the ever-growing list of inflammasome-driven disorders proves [76]. In this regard, basket trials, which study one therapy across multiple disease states, may be especially applicable [37, 77]. Currently, the most promising areas of investigation for RRx-001 are in cancer, specifically SCLC, acute myelogenous leukemia (AML) and myelodysplastic syndrome, and hepatocellular carcinoma (HCC), as well as ARS, SOM, and neurodegenerative diseases. In addition, based on preclinical data, cardiopulmonary indications seem especially well-suited for RRx-001 treatment given how the molecule mediates the release of nitric oxide under hypoxia to counter endothelial dysfunction and increased blood flow [78, 79].

A related issue for inflammasome inhibitors is how to market them in different indications. By convention, pharmaceuticals are priced uniformly, according to dosing unit, regardless of the indication for which they are prescribed; this incentivizes the use of higher doses (and longer courses of therapy) for new indications and, conversely, disincentivizes the use of lower doses (and shorter courses of therapy). A case in point is Avastin (bevacizumab, Genentech), a monoclonal antibody that targets vascular endothelial growth factor (VEGF). Avastin is approved for intravenous use only as a cancer therapy, despite evidence of “off-label” intravitreal activity for age-related macular degeneration (AMD). In AMD, where required doses are small, for example, 1.25 mg in 50 μL, Avastin costs US$17–US$50 per injection. However, in cancer, where doses 40 times larger are given, the cost (before Avastin went off patent) was US$100,000 per patient per year. This motivated Genentech to develop and market ranibizumab (Lucentis) in place of Avastin in AMD. Now generic, Lucentis is another anti-VEGF with equivalent activity to Avastin, but with, at the time, before patent expiry, a non-equivalent price of US$2000 per injection [80, 81].

Different than Avastin, RRx-001 is used at lower doses in cancer, normally a terminal disease, and higher doses elsewhere. In oncology, at lower doses, but not at higher ones, RRx-001 normalizes the tumor vasculature, which improves delivery of chemotherapy and oxygen for chemo- and radiosensitization [43]. However, for inflammatory diseases, doses are higher (and treatment courses are longer than in cancer) to fully inhibit the NLRP3 inflammasome. This supports the pursuit of non-cancer indications. In addition, it is possible to administer RRx-001 either intravenously or by mouth or subcutaneously, depending on the disease and the acuteness and severity of the symptoms—this is another differentiating factor. For immediate onset of action and complete bioavailability, intravenous administration is preferred, as in cancer, while for self-sufficiency and convenience, oral or subcutaneous administration is better, especially for the treatment of more chronic diseases like Alzheimer’s disease, where travel and mobility limitations are present.

To date, RRx-001 is well-tolerated and no dose-limiting toxicities and no drug–drug interactions have been observed. For this reason, RRx-001 could lend itself to combination with multiple other agents. Like in cancer, where RRx-001 not only increases perfusion and the delivery of chemotherapy and immunotherapy but also reduces their side effects, RRx-001 may increase the efficacy and delivery to inflamed areas of other anti-inflammatory agents like IL-6 inhibitors such as tocilizumab and sarilumab, IL-1 inhibitors such as anakinra, rilonacept, and canakinumab, and conventional disease-modifying antirheumatic drugs (DMARDs) such as methotrexate, sulfasalazine, leflunomide, and hydrochloroquine.

Finally, it has been stated that “all roads lead to inflammation” [82]. This is certainly the case for RRx-001, which en route to its ultimate, hoped-for destination, regulatory approval, as an anticancer agent, was discovered to treat a range of inflammatory diseases in preclinical models both in and outside of cancer as an NLRP3 inflammasome inhibitor. These indications and the specific mechanisms that RRx-001 may target for disease modification are being explored in ongoing research programs.

**Table 1:** Pharmacological properties and pathways targeted by RRx-001 (bromonitrozidine) therapy

Target pathway	IC_50_	In vitro studies	Key animal studies	References
NLRP3	116.9 nM	RRx-001 dose-dependently blocks IL-1β secretion, caspase-1 cleavage, and cell death at concentrations of 100–300 nM bone marrow derived macrophages. Noncanonical NLRP3 inflammasome activation triggered by cLPS was also suppressed by RRx-001	Studies from multiple groups have confirmed that RRx-001 significantly inhibits levels of caspase-1 and/or IL-1β levels and/or IL-18 levels in animal models of LPS-induced systemic inflammation, colitis, EAE, allergic asthma, Alzheimer’s disease, and non-alcoholic steatohepatitis	[40, 74, 72]
Nrf2	5 μM	In CACO2 cells, RRx-001 treatment promoted accumulation of Nrf2 in the nucleus	At 8 h post-irradiation, RRx-001-treated animals showed a significant increase in the expression of serum HO-1, one of the genes regulated by Nrf2, compared to non-RRx-001-treated animals	[83]
NF-κB	< 1 μM	In NF-κB reporter engineered BV2 cells, RRx-001 pretreatment significantly reduced NF-κB transcriptional activity	Not yet confirmed in vivo	–
CD47	2 μM	RRx-001 downregulates the expression of CD47 on tumor cells and stimulates tumor cell phagocytosis	In tumor bearing mice, depletion of macrophages by clodronate reduces the antitumor effects of RRx-001	[83]
VEGF	< 1 μM	In human vascular endothelial cells, RRx-001 significantly decreased capillary-like tube structure formation in Matrigel angiogenesis assays	As evidence of vascular normalization in tumors, pretreatment with RRx-001 of animals bearing human glioblastoma xenografts increased the concentration of irinotecan and IV and PO temozolomide in the tumor tissue. The combination groups also had statistically significant greater survival compared to the cytotoxic drug and RRx-001 alone and against control	[84]

*cLPS* cytosolic lipopolysaccharide, *EAE* experimental autoimmune encephalomyelitis, *HO-1* heme oxygenase-1, *IC50* half-maximal inhibitory concentration, *IL* interleukin, *IV* intravenous, *LPS* lipopolysaccharide, *LRR* leucine-rich repeats, *NF-κB* nuclear factor-κB, *NLRP3* NOD-, LRR-, and pyrin domain-containing 3, *NOD* nucleotide-binding domain, *Nrf2* nuclear factor erythroid 2-related factor 2, *PO* by mouth, *VEGF* vascular endothelial growth factor

**Table 2:** Disease indications where RRx-001 has demonstrated activity

Disease category	Indication	Status	Results	References
Chemo and radioprotection	Mucositis	Phase 2	Dramatic improvement in severity, time to onset, duration, and resolution compared to control	[85]
	Renal and myeloprotection	Preclinical	Statistically significant protection with cisplatin vs. cisplatin alone	[62]
	GI radioprotection	Preclinical	Significantly protected intestinal crypt cells vs. control	[86]
	Survival after lethal irradiation	In development for FDA Animal Rule	Significantly prolonged survival vs. control	[87]
	GI chemoprotection	Phase 2	Dramatically reduced incidence and severity of chemo-induced diarrhea	[66]
	Cardioprotection	Preclinical	Significant cardioprotection against doxorubicin vs. control	[88]
Neuroprotection	Alzheimer’s disease	Preclinical	Disease modification in Alzheimer’s disease models and preserved cognitive capacity vs. control animals	
	Multiple sclerosis	Preclinical	Reversed clinical course and significantly decreased central nervous system inflammatory infiltration and demyelination in the spinal cord vs. controls	[47]
Fibrosis/metabolic	NASH	Preclinical	Reduced hepatic steatosis scores, serum transaminase levels, and hepatic levels of cholesterol and triglycerides in rats fed a high-fat diet	[89]
Autoimmune	Inflammatory bowel disease	Preclinical	Decreased colitis vs. control in an induced mouse model	[50]
Cardiopulmonary	Ischemia reperfusion injury	Preclinical	RRx-001 prevents or attenuates ischemia reperfusion injury	[69]
	Hemorrhagic shock	Preclinical	RRx-001 restores microvascular perfusion	[90]
	Cerebral malaria	Preclinical	RRx-001 treatment decreases mortality both alone and in combination with artemether	[91]
	Asthma	Preclinical	RRx-001 significantly reduced inflammatory cell infiltration and mucus secretion in the airway in an inhaled house dust mite model of asthma	[72]
	Pulmonary hypertension	Clinical trial planned	RRx-001 significantly attenuated right heart failure and vessel wall thickening vs. control	[91]
	Exercise enhancement	Preclinical	RRx-001 treated animals tolerate high-intensity exercise significantly better than non-RRx-001 treated animals	[68]

*GI* gastrointestinal, *FDA* Food and Drug Administration, *NASH* non-alcoholic steatohepatitis

## Declarations

### Availability of data and materials

Data sharing is not applicable to this article as no datasets were generated or analyzed during the current study.
